# Altar of Domestic Dirt

**Published:** 1920-08-14

**Authors:** 


### Altar of Domestic Dirt.

The question as to where milk in the process of its production, transport, and supply to babies is most contaminated is raised in a pamphlet written by Mr. Ben Davies, of Portman Square, on the subject of proposed milk legislation. His view is that the apportionment of blame for contamination, as indicated by the Milk and Dairies Bill, is not accurate, and he says that though the source of milk should not be neglected, the gravest dangers are encountered after the milk leaves the farm. He writes:

We have it on the authority of Sir George Newman and Sir Arthur Newsholme that the most serious single cause of infant mortality is due not to farm contamination of milk, but to its domestic infection, and it begins to appear that the two next most serious causes of infant deaths—viz., bronchitis and pneumonia—are due to the same condition. Yet when we come to a draft Bill to save baby life—for a Milk Bill with a lesser object would be a fraud—we close our eyes to evidence which has been accumulating for a third of a century, and which one of the greatest authorities in the land declares is "overwhelming," and concentrate on farm inspection merely to eliminate things we now know are not serious causes of trouble. To devise an organisation to save the possible one bovine tuberculous baby and do nothing and attempt nothing to save the 20,000 babies who at the same time are immolated on the altar of homely, domestic, human dirt, is an extraordinary instance of refusal to abandon a discredited theory, and of deliberate rejection of scientific knowledge and experience.

It is good to have this point of domestic cleanliness driven home, because no amount of legislation imposed upon the dairy farmer and the distributing agencies can have complete success without the co-operation of the homes. On the other hand it seems to us that it is beginning at the right end first to secure that the milk shall be delivered in the cleanest possible condition. By all means let the urgency of cleanliness in the home be simultaneously emphasised, but the housewife must first be given something clean if she is to keep it clean.

